# Synergistic Effects of Rhizobacteria and Salicylic Acid on Maize Salt-Stress Tolerance

**DOI:** 10.3390/plants12132519

**Published:** 2023-06-30

**Authors:** Qasim Ali, Maqshoof Ahmad, Muhammad Kamran, Sana Ashraf, Muhammad Shabaan, Babar Hussain Babar, Usman Zulfiqar, Fasih Ullah Haider, M. Ajmal Ali, Mohamed S Elshikh

**Affiliations:** 1 Department of Soil Science, Faculty of Agriculture and Environment, The Islamia University of Bahawalpur, Bahawalpur 63100, Pakistan; ali.qasim@iub.edu.pk (Q.A.); maqshoof_ahmad@yahoo.com (M.A.); 2 Pakistan Council for Science and Technology, Ministry of Science and Technology, Islamabad 44000, Pakistan; hafizkamran1576@gmail.com; 3 College of Earth and Environmental Sciences, Quaid-e-Azam Campus, University of the Punjab, Lahore 54590, Pakistan; sanashrafpu@gmail.com; 4 Land Resources Research Institute (LRRI), National Agricultural Research Centre (NARC), Islamabad 44000, Pakistan; mshabaan@parc.gov.pk; 5 Vegetable and Oilseed Section, Agronomic Research Institute, Faisalabad 38850, Pakistan; babarhbabar@gmail.com; 6 Department of Agronomy, Faculty of Agriculture and Environment, The Islamia University of Bahawalpur, Bahawalpur 63100, Pakistan; 7 Key Laboratory of Vegetation Restoration and Management of Degraded Ecosystems, South China Botanical Garden, Chinese Academy of Sciences, Guangzhou 510650, China; 8 University of Chinese Academy of Sciences, Beijing 100039, China; 9 Department of Botany and Microbiology, College of Science, King Saud University, Riyadh 11451, Saudi Arabia; alimohammad@ksu.edu.sa (M.A.A.); melshikh@ksu.edu.sa (M.S.E.)

**Keywords:** PGPR, salicylic acid, malondialdehyde, ascorbate peroxidase, relative water contents

## Abstract

Maize (*Zea mays* L.) is a salt-sensitive plant that experiences stunted growth and development during early seedling stages under salt stress. Salicylic acid (SA) is a major growth hormone that has been observed to induce resistance in plants against different abiotic stresses. Furthermore, plant growth-promoting rhizobacteria (PGPR) have shown considerable potential in conferring salinity tolerance to crops via facilitating growth promotion, yield improvement, and regulation of various physiological processes. In this regard, combined application of PGPR and SA can have wide applicability in supporting plant growth under salt stress. We investigated the impact of salinity on the growth and yield attributes of maize and explored the combined role of PGPR and SA in mitigating the effect of salt stress. Three different levels of salinity were developed (original, 4 and 8 dS m^−1^) in pots using NaCl. Maize seeds were inoculated with salt-tolerant *Pseudomonas aeruginosa* strain, whereas foliar application of SA was given at the three-leaf stage. We observed that salinity stress adversely affected maize growth, yield, and physiological attributes compared to the control. However, both individual and combined applications of PGPR and SA alleviated the negative effects of salinity and improved all the measured plant attributes. The response of PGPR + SA was significant in enhancing the shoot and root dry weights (41 and 56%), relative water contents (32%), chlorophyll a and b contents (25 and 27%), and grain yield (41%) of maize under higher salinity level (i.e., 8 dS m^−1^) as compared to untreated unstressed control. Moreover, significant alterations in ascorbate peroxidase (53%), catalase (47%), superoxide dismutase (21%), MDA contents (40%), Na^+^ (25%), and K^+^ (30%) concentration of leaves were pragmatic under combined application of PGPR and SA. We concluded that integration of PGPR and SA can efficiently induce salinity tolerance and improve plant growth under stressed conditions.

## 1. Introduction

With the world’s population estimated to reach 9.6 billion by 2050, agricultural output must be increased to feed an additional 2 billion people by that year [1]. However, salinization of arable land may impede the globe’s ability to maintain a steady food supply for its growing population [2]. Salinity stress decreases yields of essential cereal crops such as maize, rice, and wheat yearly [3,4,5,6]. Irrigated land is responsible for producing one third of the world’s food supply. It is severely affected by salt damage upto 20% (45 million ha), which will expand to more than 40% of the global irrigated agricultural area in future [7]. In Pakistan, salinization has made 8.6 million hectares (mha) of agricultural lands unusable due to its arid to semiarid climate [8,9]. Excessive salt concentrations harm soil’s physicochemical and biological properties [10], affecting the activity of soil microbes and plant roots, and ultimately hampering crop growth and yields [11].

Managing crop productivity on saline soils will be the most complex and challenging task in the upcoming years due to the ionic and osmotic stresses faced by plants under exposure to salinity, leading to changes in their morphology, physiology, and biochemistry [12,13]. Salinity harms the membranes, reduces the efficiency of photosynthesis, disrupts nutrient balance, alters the levels of growth regulators, inhibits enzyme activity, and causes metabolic dysfunction [14]. Furthermore, salt toxicity induces ion-specific stress in plant cells, altering the K^+^/Na^+^ ratio [15]. Additionally, detrimental effects of salinity increase the production of reactive oxygen species (ROS) by disrupting the redox balance of plant cells, causing damage to essential molecules and organelles within the cells [16]. Plants have a natural defense system that includes limiting the uptake of toxic ions, storing toxic ions in vacuoles, producing osmoprotective compounds, and regulating stomata to maintain water levels in salt-stressed plants generating both enzyme-based and non-enzyme-based antioxidants [17,18,19,20]. However, when salinity stress is severe, plants cannot protect themselves as effectively, and are more susceptible to the damage caused by salt. The quest for suitable techniques to induce salinity tolerance in plants continues, ultimately aiding them in combating the harmful effects of salt stress [21]. Salicylic acid (SA) is a phenolic compound that acts as a growth regulator in a wide range of physiological and biochemical processes, including plant growth, thermogenesis, flowering and ion uptake [22,23]. SA treatment also reduces lipid peroxidation, and it has the potential to interact with other plant hormones to make plants more resistant to the effects of salt stress [24]. Exogenous application of SA has demonstrated tremendous potential to improve salt tolerance in various plant species [24,25,26]. By regulating stomatal behavior and hormonal status and increasing osmolytes, antioxidants, proteins, and secondary metabolites, SA treatment alleviates the harmful effects of salinity [20,27]. Moreover, it can function as an antioxidant, scavenging accumulated ROS [28]. SA treatment improves plant water status, nutritional uptake, and biomass, favoring the production of essential oils in medicinal and aromatic plants [25].

Plant growth-promoting rhizobacteria (PGPR) dwell in the rhizosphere, and support the healthy growth of plants by adopting various mechanisms related to plant growth promotion. Root exudates contain free amino acids, carbohydrates, vitamins, and other elements necessary for the growth of rhizobacteria [29,30]. PGPR are involved in various processes that contribute to the growth and development of plants. They produce a range of phytohormones that promote plant growth and fix atmospheric nitrogen (N). Additionally, PGPR contain enzymes responsible for mineral solubilization that regulates plant growth [31,32].

Maize is an important grain crop that can be grown in various climates [33]. It is important worldwide due to its versatility and adaptability to different genotypes, soils, and climates [34]. Although, maize is moderately sensitive to salt, the interplay between SA and salt-tolerant PGPR creates a feedback loop that enhances stress resilience. However, many unanswered questions remain regarding their behavior under salt stress. Despite the vigorous research being executed in exploiting the role of plant growth regulators (PGRs) and PGPRs in ameliorating the salinity stress in maize, much research is needed regarding the role of SA and salt-tolerant PGPR in alleviating the role of salt stress in plants. Therefore, present study aimed to investigate the potential benefits of administering exogenous SA in combination with salt-tolerant PGPR to mitigate the harmful effects of NaCl stress on growth, yield, ionic content, and antioxidant defense of maize plants grown under salinized soil. We hypothesized that combined application of PGPR and SA could uplift the antioxidative plant defense system, and regulate its physiological activities. In this way, it can sustain plant growth under stressed conditions by improving its growth and yield. 

## 2. Results

### 2.1. Effects on Shoot and Root Attributes of Maize

**Figure 1:**
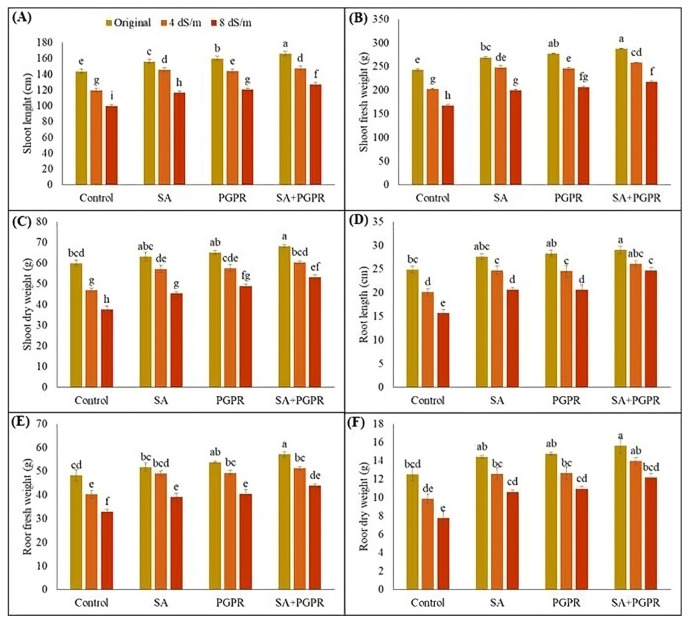
Effect of combined application of PGPR and SA on (**A**) shoot length (cm), (**B**) shoot fresh weight (g), (**C**) shoot dry weight (g), (**D**) root length (cm), (**E**) root fresh weight (g), and (**F**) root dry weight (g) of maize under saline conditions. Error bars show the standard deviation from three independent replications. Variations in lowercase show significant difference between PGPR and SA treatments at *p* ≤ 0.05. SA: salicylic acid; PGPR: salt-tolerant rhizobacterial strain; SA + PGPR: combination.

Present study observed that maize’s root and shoot attributes were adversely affected by salt stress, and impact was more pronounced at higher salt concentrations than lower levels. However, applying rhizobacterial inoculation and SA significantly improved root and shoot attributes of maize under saline conditions. Furthermore, combined application of PGPR and SA proved more effective than their sole applications (Figure 1). Compared to control, a significant increase in root and shoot length of maize was observed due to the combined effect of PGPR and SA, with a 57% and 27% increase under 8 dS m^−1^ and a 30% and 23% increase under 4 dS m^−1^ salinity levels, respectively. Among their sole application, PGPR resulted in more noticeable results than SA in improving maize’s shoot and root length under both normal and saline conditions.

Regarding root and shoot fresh weights, a significant increment of 34 and 30% was recorded due to the combined application of PGPR and SA under 8 dS m^−1^. Similarly, combined treatment (PGPR + SA) improved root and shoot fresh weight by 27 and 28%, respectively, compared to their respective uninoculated and untreated control under 4 dS m^−1^. Additionally, individual and combined treatment of PGPR and SA enhanced the dry weight of maize, with increases ranging from 5 to 21%, 9 to 30%, and 14 to 41% for shoot and 15 to 36%, 18 to 41% and 25 to 56% for root, respectively. Highest increase in dry shoot weight of maize (41% higher than the respective control) was observed in PGPR + SA treated plants under 8 dS m^−1^ of salt level as compared to PGPR (30% higher than the respective control) and SA (21% higher than the respective control) alone. Similarly, highest root dry weight due to PGPR +SA was observed under 8 dS m^−1^ as compared to PGPR and SA alone (Figure 1).

### 2.2. Effect on Maize Yield and Yield-Related Attributes and Relative Water Contents

**Figure 2:**
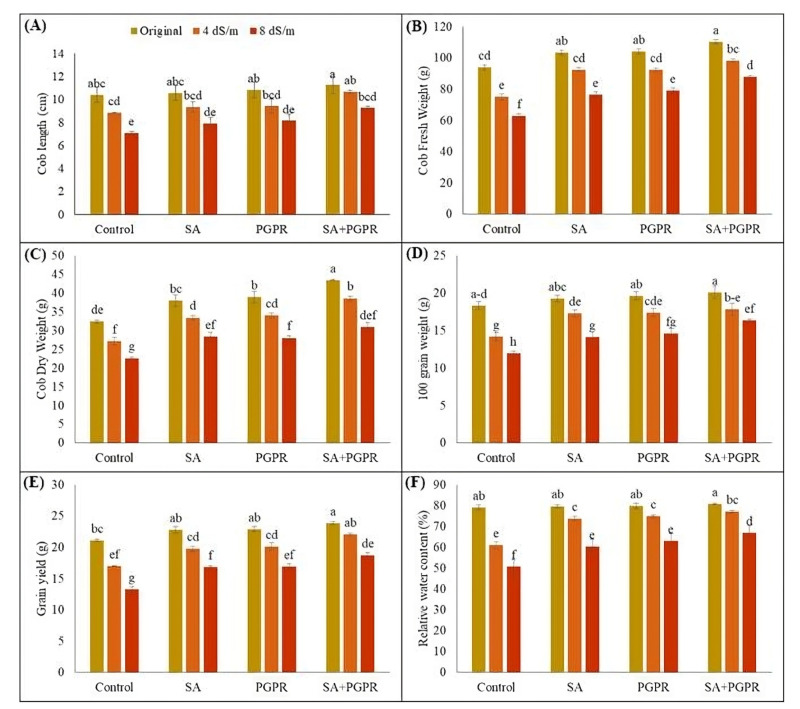
Effect of combined application of PGPR and SA on (**A**) cob length (cm), (**B**) cob fresh weight (g), (**C**) cob dry weight (g), (**D**) 100 grain weight (g), (**E**) grain yield (g), and (**F**) relative water content (%) of maize under saline conditions. The error bars show the standard deviation from three independent replications. Variations in lowercase show significant difference between PGPR and SA treatments at the *p* ≤ 0.05. SA: salicylic acid; PGPR: salt-tolerant rhizobacterial strain; SA + PGPR: combination.

Salinity stress negatively impacted yield-related parameters of maize under different salt concentrations (Figure 2A–E). As concentration of NaCl increased from 4 to 8 dS m^−1^, there was a noticeable decrease in cob length, fresh and dry weight of cob, 100-grain weight, and maize yield. However, applying PGPR, SA, and PGPR + SA significantly improved all the yield-related parameters under normal and salt-stressed conditions. Under normal conditions, PGPR, SA, and PGRP + SA increased the shoot length (2, 4 and 8%), fresh weight (10, 11 and 18%), and dry weight (17, 20 and 34%) of maize cob as compared to respective control. At 4 dS m^−1^, highest increase of 21, 31, and 42% in shoot length, fresh and dry cob weight was observed with the application of PGPR + SA as compared to the respective control. Similarly, at 8 dS m^−1^, maximum increase of 31, 39 and 37% in shoot length, fresh and dry weights of cob was recorded with the application of PGPR + SA as compared to respective uninoculated untreated stressed control. Salt stress also affected grain yield and 100 grain weight that were significantly improved by applying PGPR, SA, and their combination at all salt concentration levels. Highest increase in grain yield and 100-grain weight (41 and 37% higher than control) were recorded when maize plants were treated with PGPR + SA at 8 dS m^−1^. Individual application of PGPR and SA had statistically similar effects in improving the grain yield, and caused 27% increase as compared to respective control at 8 dS m^−1^. Regarding 100 grain weight, PGPR inoculation had a statistically similar effect to the other treatments, with 22% increase as compared to control (Figure 2A–E). Furthermore, salt stress led to a significant reduction in RWC by 23 and 36% at 4 and 8 dS m^−1^ NaCl as compared to non-saline control. Applying PGPR, SA and PGPR + SA improved the RWC of maize by 21, 23, and 26% at 4 dS m^−1^ and by 19, 24, and 32% at 8 dS m^−1^, respectively, as compared to respective untreated control (Figure 2F).

### 2.3. Effects on Photosynthesis Pigments and Ionic Content of Maize Plants

**Figure 3:**
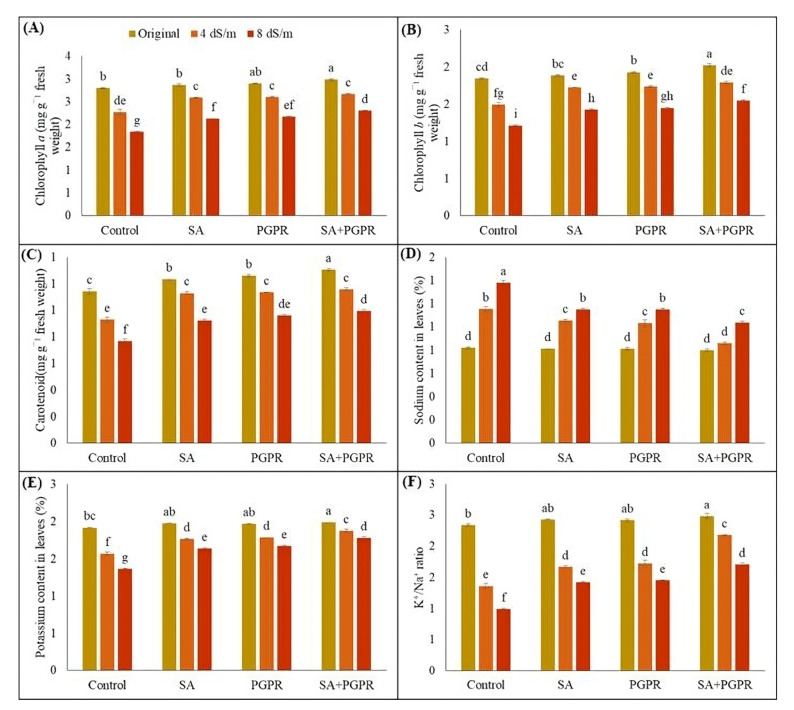
Effect of combined application of PGPR and SA on (**A**) chlorophyll a (mg g^−1^ f. wt.), (**B**) chlorophyll b (mg g^−1^ fresh weight.), (**C**) carotenoid (mg g^−1^ fresh weight.), (**D**) sodium content in leaves (%), (**E**) potassium content in leaves (%), and (**F**) K^+^/Na^+^ ratio of maize under saline conditions. Error bars show the standard deviation from three independent replications. Variations in lowercase show significant difference between PGPR and SA treatments at the *p* ≤ 0.05. SA: salicylic acid; PGPR: salt-tolerant rhizobacterial strain; SA + PGPR: combination.

Figure 3 illustrates the effect of NaCl-induced salt stress on photosynthetic pigments and ionic content of treated and untreated maize. It was observed that PGPR, SA and their combination enhanced the chlorophyll a, b and carotenoid contents under both normal and saline conditions. At 8 dS m^−1^, highest increase in chlorophyll a content was observed in the treatment, where PGPR and SA were used together (25%) as compared to sole applications of PGPR (18%) and SA (15%). Similarly, compared to respective control at 8 dS m^−1^ salinity, combined treatment of PGPR and SA increased chlorophyll b by 127% than PGPR (20%) and SA (17%) alone. Similar trends were observed in case of carotenoids with a maximum increase of 30% due to combined application of PGPR and SA than respective control at 8 dS m^−1^ salinity level (Figure 3A–C).

Salinity stress negatively affected K^+^, Na^+^ concentrations and K^+^/Na^+^ ratio in treated and untreated maize plants, (Figure 3D–F). Specifically, salinity stress increased the Na^+^ concentration while decreasing the K^+^ concentration and the K^+^/Na^+^ ratio at all salt levels. At 8 dS m^−1^, Na^+^ concentration in leaves of untreated plants increased by 68% as compared to unstressed control, which resulted in a significant decrease in K^+^ concentration and K^+^/Na^+^ ratio by 28.6 and 57.69%, respectively. However, applying PGPR, SA and their combined treatment significantly reduced the Na^+^ concentration in leaves by 17, 17 and 25%, respectively. Contrastingly, it increased the K^+^ concentration by 20, 22, and 30% and the K^+^/Na^+^ ratio by 44, 47 and 72%, respectively, as compared to untreated control at 8 dS m^−1^. Individual application of PGPR and SA did not show significant differences in improving Na^+^, K^+^ concentrations and K^+^/Na^+^ ratio in leaves of maize under 8 dS m^−1^ of salt level. At 4 dS m^−1^, combined treatment of PGPR and SA resulted in a 25% decrease in Na^+^ concentration, and a 19 and 16% increase in K^+^ concentration and K^+^/Na^+^ ratio in leaves of maize respectively, as compared to respective control. The PGPR and SA treatment remained statistically non-significant to each other with a 17% decrease in Na^+^ concentration each, a 20 and 22% increase in K^+^ concentration, and a 44 and 47% increase in K^+^/Na^+^ ratio of leaves than respective control at 4 dS m^−1^ (Figure 3D–F).

### 2.4. Effects on Antioxidant and Oxidant System of Maize Plant

**Figure 4:**
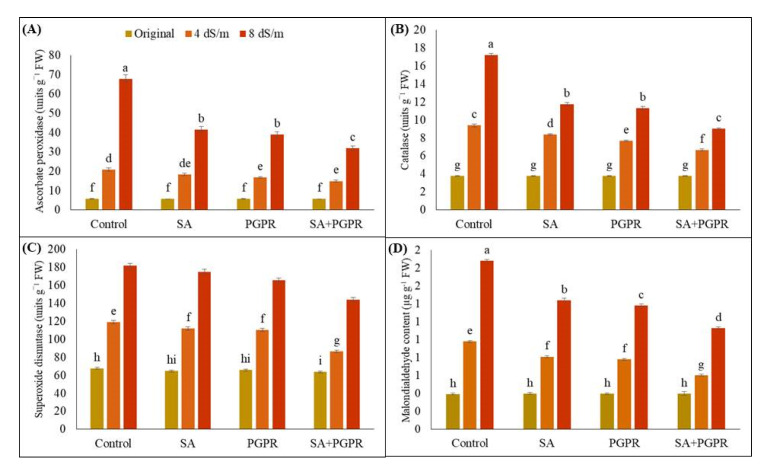
Effect of combined application of PGPR and SA on (**A**) ascorbate peroxidase (units g^−1^ FW), (**B**) catalase (units g^−1^ FW), (**C**) superoxide dismutase (units g^−1^ FW), and (**D**) malondialdehyde content (µg g^−1^ FW) of maize under saline conditions. The error bars show the standard deviation from three independent replications. Variations in lowercase show statistical significance between PGPR and SA treatments at the *p* ≤ 0.05. SA: salicylic acid; PGPR: salt-tolerant rhizobacterial strain; SA + PGPR: combination.

Figure 4 depicts the changes in APX, CAT, SOD and MDA levels in response to salinity stress. Maize plants treated with higher concentrations of NaCl (4 and 8 dS m^−1^) exhibited elevated production of APX, CAT, SOD, and MDA in their leaves as compared to the untreated maize plants under salt stress. However, significant reductions were observed in APX levels due to PGPR, SA and PGPR + SA, which were 39, 43, and 53%, respectively, as compared to respective untreated control at 8 dS m^−1^ salt concentration. At 4 dS m^−1^, application of PGPR, SA, and PGPR + SA resulted in a decrease in APX activities by 12, 19, and 28%, respectively, as compared to untreated control (Figure 4A). For CAT, application of PGPR, SA, and their combination resulted in 32, 39 and 47% decrease at 8 dS m^−1^ salinity level.

Similarly, under the influence of salt stress (4 dS m^−1^), introduction of PGPR, SA, and their combination (PGPR + SA) led to a reduction of 11, 18 and 29% respectively, in maize CAT (catalase) contents as compared to respective control (Figure 4B). Most significant decline in SOD (superoxide dismutase) contents in maize leaves was observed with the combined application of PGPR + SA at 8 dS m^−1^, resulting in a 21% decrease as compared to control. When applied individually, PGPR and SA caused a decrease of 4 and 9%, respectively, in SOD content of maize compared to the control. Notably, at 4 dS m^−1^, combined application of PGPR and SA exhibited the most substantial increase in SOD content as compared to respective control. Furthermore, PGPR and SA, when applied separately, demonstrated similar effects on the SOD content of maize, causing 6 and 7% decrease, respectively, at 4 dS m^−1^ (Figure 4C). In terms of MDA (malondialdehyde) contents, application of PGPR and SA, whether alone or in combination, resulted in a significant reduction. Combined application of PGPR + SA exhibited the most substantial decrease in MDA content at 8 dS m^−1^, demonstrating a 38% decrease compared to control at 4 dS m^−1^ (Figure 4D).

### 2.5. Pearson Correlation Analysis

**Figure 5:**
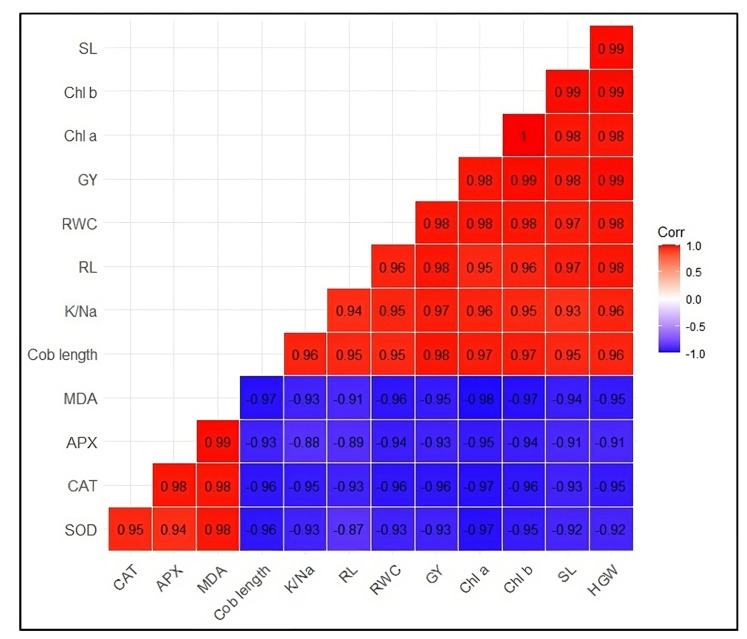
Correlation matrix SL (shoot length), chl a (chlorophyll a), chl b (chlorophyll b), GY (grain yield), RWC (relative water content), RL (root length), K/Na (K^+^/Na^+^ ratio), cob length, MDA (malondialdehyde), APX (ascorbic peroxidase), CAT (catalase), SOD (Superoxide dismutase), HGW (100 grain weight).

Pearson correlation (Figure 5) results indicated a strong correlation between different plant growth and yield attributes and antioxidant activities. However, correlation was strongly negative. It was observed that CAT activities were strongly correlated with shoot length (r = 0.93), chlorophyll ‘a’ and ‘b’ contents (r = 0.97 and 0.96), cob length (r = 0.96), K/Na ratio (r = 0.95) and grain yield (r = 0.96). Similarly, APX activities were also observed to have a strong negative correlation with shoot length (r = 0.91), chlorophyll ‘a’ and ‘b’ contents (r = 0.95 and 0.94), cob length (r = 0.93), K/Na ratio (r = 0.88) and grain yield (r = 0.93). Moreover, SOD activities in maize leaves were also strongly correlated with shoot length (r = 0.92), chlorophyll ‘a’ and ‘b’ contents (r = 0.97 and 0.95), cob length (r = 0.96), K/Na ratio (r = 0.93) and grain yield (r = 0.93).

## 3. Discussion

Current study found that salinity stress had a detrimental effect on plant growth, specifically in shoot length, root length, and biomass. The negative impact of salinity treatment is more pronounced at higher salinity levels. Previous studies have also reported decreased growth and a decline in biomass across various plant species due to soil salinity [35,36,37,38]. Growth retardation has been identified as a significant factor determining the extent of damage caused by salinity in different plant species [17]. Furthermore, Ahmad et al. [39] observed that maize cultivars are particularly susceptible to salinity-induced damage. Reduced growth, biomass, and yield in maize plants primarily result from the interruption in cell division and elongation caused by salt stress. In this study, exogenous application of two protective agents, PGPR and SA, in mitigating the adverse impacts of salinity stress on plant growth were assessed. Both protectants are well-known for their potential to enhance crop growth. When applied exogenously, both PGPR and SA resulted in improved growth and increased biomass output in the presence of salt stress. Similar findings were reported by Aneela et al. [40] and Khan et al. [41], who observed increased growth even under salt-stress conditions after applying PGPR or SA.

The superiority of PGPR over SA can be attributed to the dose-dependent suppression of auxin under SA treatment, as reported by Pasternak et al. [42]. PGPR exhibits its efficacy in promoting plant growth and development through various mechanisms. These include enhanced nutrient assimilation, biological nitrogen fixation, phosphorous solubilization, and iron acquisition [43,44]. Moreover, PGPR aids pathogen control via antagonism and competition [44,45,46]. Application of PGPR has been discovered to impact the management of abiotic stress through both direct and indirect mechanisms, ultimately resulting in the development of systemic tolerance [47,48].

The levels of photosynthetic pigments, crucial for efficient energy absorption in plants are significantly influenced by environmental conditions [49]. During current investigation, it was noted that exposure to salt stress led to a decrease in the levels of photosynthetic pigments, which can resultantly cause reduction in energy assimilation and carbohydrate production ultimately, hampering biomass production in salt-stressed plants. Numerous studies have demonstrated the effectiveness of PGPR and SA as regulators of photosynthesis. They positively influence the structure of the chloroplasts and leaves and the levels of chlorophyll and carotenoids [50,51]. Our results align with these findings, as we observed increased photosynthetic pigment levels in salt-stressed plants after applying PGPR and SA. SA not only regulates photosynthesis but also contributes to chloroplast formation. It protects chloroplasts from ROS, enhances chlorophyll stability, and helps maintain photosynthetic pigments under salt stress. In contrast, PGPR inoculation promotes higher photosynthetic pigment production by improving mineral absorption [52].

Mineral absorption in plants is a necessary adaptation for stress tolerance [53]. Due to their similar physicochemical structures, Na^+^ and K^+^ compete for uptake [54]. In our study, salt stress induced Na^+^ uptake while decreasing plant K^+^ levels, resulting in a lower K^+^/Na^+^ ratio. Ahmad et al. [55] also documented similar findings in mustard plants and Karlidag et al. [56] in strawberries. Ahmad et al. demonstrated that increasing NaCl concentration led to increased uptake of Na^+^ while decreasing the uptake of K^+^ and Ca^2+^ [55]. It was also found that soybean cultivars that accumulate lower levels of Na^+^ and higher levels of K^+^ exhibit greater resistance to salinity stress. Under salt stress, plants accumulate higher Na^+^, Cl−, SO_4_^2−^, and Ca^2+^ ions, leading to severe ion toxicity [57]. Furthermore, the toxicity caused by a particular ion varies among plant species [17,58], and it may disrupt cytoplasmic metabolic processes and negatively affect photosynthesis [59]. In our investigation, NaCl-induced stress increased Na^+^ uptake, decreased K^+^/Na^+^ ratio, and reduced K^+^ absorption. Similar results regarding the increase in Na^+^ and decrease in K^+^/Na^+^ ratio and K^+^ by El-Katony et al. [60] in maize and Cai and Gao [61] in quinoa (*Chenopodium quinoa* (Willd.)) have been reported. The competitive nature of Na^+^ and K^+^ for absorption, owing to their similar physicochemical composition, was emphasized by Ahanger and Agarwal [62]. In addition, Ahmad et al. [63] demonstrated that increasing NaCl concentration enhanced Na^+^ uptake, consequently reducing the amount of K^+^ absorbed by the cell. Ultimately, efficient mineral uptake is considered one of the primary adaptations for stress resistance [53].

Application of SA and PGPR resulted in an increased K^+^ uptake, and this effect can be attributed to SA-induced H^+^-ATPase activity and production of exopolysaccharides by PGPR [64,65]. PGPR produces exopolysaccharides that bind to Na^+^, and reduces its availability. The connection between SA and intake of mineral nutrient is associated with SA’s role in mitigating salt stress [66]. PGPR enhanced K^+^ uptake while reducing Na^+^ uptake to a greater extent than SA. Studies have shown that PGPR can improve the exchange of both macro and micronutrients, rectifying the nutrient imbalance caused by excessive Na^+^ and K^+^ input. The role of SA in alleviating the adverse effects of salt stress is likely linked to its influence on mineral nutrient uptake [64,67]. Plant nutrient availability can be improved through various means, such as microbial-mediated nutrient cycling (mineralization), alterations in rhizosphere pH (via organic acids), and metal chelation (using siderophores) [64,67]. PGPR plays a crucial role in regulating ion homeostasis, and maintaining high K^+^/Na^+^ ratios in plant shoots. It achieves this by reducing the accumulation of Na^+^ and Cl− in leaves, facilitating the faster exclusion of Na^+^ through the roots, and enhancing the activity of high-affinity K^+^ transporters [67].

RWC is considered a valuable indicator for estimating plant water contents, considering metabolic processes occurring in plant tissues. Salinity stress often disrupts the water balance in plants. Reduction in RWC observed under salinity conditions can be attributed to the shrinkage of root systems, which cannot adequately compensate for water loss caused by the reduced absorbing surface area due to transpiration [68]. However, application of PGPR and SA resulted in increased root growth, leading to higher RWC, and improved metabolic activity in maize plants as examined in this study.

In present study, salinity stress resulted in an increased content of MDA in maize plants. Various researchers have attributed the elevated MDA levels to increased production of ROS under salt stress [69,70,71]. MDA, a byproduct of lipid peroxidation, directly reflects the extent of damage caused by salt stress in plants [72]. Moustafa-Farag and colleagues [73] found that elevated MDA levels during salt stress resulted from increased ROS generation, which enhanced oxidative stress in plant tissues. Accumulation of MDA results from a cascade of oxidative damage triggered by increased oxidative stress. However, exogenous application of PGPR and SA mitigated lipid injury, and maintained structural integrity of membrane thereby, reducing oxidative damage caused by salt stress. 

Consequently, MDA levels in plants treated with PGPR and SA were lower than in salt-affected plants, indicating the contribution of these exogenous applications in reducing MDA levels [74]. Under normal conditions, plants maintain a dynamic balance between ROS production and degradation. However, when plants encounter salinity stress, they generate more ROS species, such as O_2_, OH, and H_2_O_2_ [75]. Plants have evolved a complex antioxidant defense mechanism to counteract excessive ROS production. This mechanism includes the action of antioxidant enzymes such as CAT, SOD, and APX [76,77]. These antioxidants directly scavenge ROS, playing a direct role in defense against abiotic stress [76,77]. In our study, applying both protectants increased the levels of CAT, SOD, and APX in plants. However, PGPR exhibited a slightly superior antioxidant activity compared to SA. Furthermore, combined application of PGPR and SA significantly reduced enzyme production, indicating the efficacy of the combined treatment in mitigating the impact of salinity stress on the induction of defense-related enzymes.

## 4. Materials and Methods

The individual and combined effects of SA and PGPR in mitigating the salinity-induced negative impacts on maize (*Zea mays* L.) were assessed in a pot experiment conducted in a wire-house of the College of Agriculture, Bahauddin Zakariya University, Layyah campus, Pakistan. Soil used in the experiment was collected from the college farm area, sieved, and used to fill each pot (12 kg). Pots were then covered with a polyethene sheet to maintain the experimental conditions.

Determination of physicochemical characteristics of soil

Experiment determined various physicochemical parameters (as shown in Table 1) following USDA Handbook No. 60 [78]. 

### 4.1. Volumetric Moisture Contents (%)

Volumetric moisture content of the soil was determined by measuring fresh and dry weights of 200 g soil samples. Samples were placed in an oven at 105 °C till they reached a constant weight. Following formula was used to calculate the volumetric moisture contents.

$$\mathrm{Volumetric}\;\mathrm{moisture}\;\mathrm{contents}\;(\%)=\frac{\mathrm{Fresh}\;\mathrm{weight}\;\mathrm{of}\;\mathrm{soil}\;(g)-\mathrm{Oven}\;\mathrm{dry}\;\mathrm{weight}\;\mathrm{of}\;\mathrm{soil}\;(g)\;}{\mathrm{Oven}\;\mathrm{dry}\;\mathrm{weight}\;\mathrm{of}\;\mathrm{the}\;\mathrm{soil}\;(g)}\times 100$$

### 4.2. Soil pH and Electrical Conductivity (EC)

The pH of saturated soil paste was measured (Ohaus pH meter model Starter 3100C), by preparing the saturated paste, and extract (ECe) of the paste was used to measure soil EC using an EC-meter (Ohaus Starter 3100C). 

### 4.3. Soil Organic Matter (OM)

Determination of SOM content involved a procedure in which soil was mixed with 10 mL of potassium dichromate (K_2_Cr_2_O_7_) and 20 mL of sulfuric acid (H_2_SO_4_). Resulting mixture was then titrated with ferrous ammonium sulfate while utilizing diphenylamine as an indicator to measure the reduction of chromic acid. Completion of reaction was indicated by the appearance of a pink color, which was subsequently, compared with a blank sample. To calculate the carbon (C) content of soil, Walkley and Black method [79] was followed. Determining the organic matter (OM) contents followed the standard practice, which involved multiplying the calculated carbon content by a factor of 1.72 [80]. 

### 4.4. Nutrients Concentration from Soil

Wet digestion of soil was carried out using concentrated H_2_SO_4_, following the method described by Gunning and Hibbard. This method involved distilling ammonia to boric acid (4%), followed by back-titration of receiver solution against 0.01N H_2_SO_4_ using the Kjeldahl apparatus. Concentration of available P in soil was determined by agitating 5 g of air-dried soil with 100 mL of 0.5M Na_2_HCO_3_ for 30 min. Contents were then filtered using Whatman filter paper # 42 [81]. Color development was achieved using ascorbic acid, and concentration of available P in the filtrate was determined using a spectrophotometer (Thermo Electron Corporation, Waltham, MA, USA) at 880 nm by comparing it with a standard curve. To determine the extractable K, soil was extracted with a 1N ammonium acetate solution, and observations were compared with standard values using a flame photometer (BWB Technologies, Newbury, UK); K involved extraction of soil followed by a comparison of observations with standard values using a flame photometer [78]. 

### 4.5. Textural Determination

Mixture for textural determination comprised of 40 g soil sample with 60 mL solution of 1% sodium hexametaphosphate, which served as a dispersing agent. Hydrometer readings were recorded after adding 150 mL of distilled water, and allowing the mixture to stand overnight. Soil texture was then determined using a textural triangle following the method described by Moodie et al. [82].

### 4.6. Surface Sterilization of Seeds

Seed surface sterilization was performed by immersing them in NaOCl (5% *w*/*v*) for one minute, followed by 2–3 rinses in distilled water. Subsequently, seeds were submerged in ethanol (70% *v*/*v*) for 30 s, and rinsed with autoclaved distilled water 5–6 times. This procedure ensured the effective sterilization of seed surface.

### 4.7. Acquisition of Salt-Tolerant PPGP Strain

Salt-tolerant *Pseudomonas aeruginosa* strain (The NCBI GenBank Accession # OP020423; https://www.ncbi.nlm.nih.gov/nuccore/OP020423.1?report=GenBank, accessed on 1 January 2020) was obtained from Soil Microbiology and Biochemistry laboratory, Institute of Soil and Environmental Sciences, University of Agriculture in Faisalabad.

### 4.8. Preparation of Inoculum

Luria Bertani (LB) broth was prepared in a 250 mL conical flask for inoculum preparation, which was later, autoclaved at 121 °C for 20 min, and cooled in a laminar flow hood. Salt-tolerant bacterial strain was inoculated to LB broth, and incubated in a shaking incubator at 28 ± 2 °C and 100 rpm for 48 h. This incubation period was necessary to achieve a uniform cell density of 10^8^–10^9^ CFU mL^−1^.

### 4.9. Seeds Inoculation

Inoculum was centrifuged at 9000× *g* for 10 min prior to seed inoculation. After this, pellets were suspended in 0.01 M MgSO_4_, and cell population was maintained at 10^8^ cells mL^−1^ using a spectrophotometer (Thermo Electron Corporation, USA). Inoculum, sterilized press mud, and 10% sugar solution in a ratio of 4:5:1 were used for seed coating. Meanwhile, seeds in control treatment were coated with a mixture of sugar solution, sterilized press mud, and sterilized broth. Coated seeds were left to air dry for 8 h under shade before sowing.

### 4.10. Pot Experiment

Experimental design consisted of four treatments, and pots containing 12 kg of soil were arranged in a factorial set with three replications using a completely randomized design (CRD). NaCl was added to maintain three salinity levels (Original, 4 and 8 dS m^−1^). First treatment served as an uninoculated untreated control whereas, second treatment involved the application of salicylic acid (SA) at a rate of 50 mg L^−1^. Third treatment consisted of PGPR inoculation, and fourth treatment combined the application of SA and PGPR strain. Five inoculated maize seeds were planted in each pot, and one healthy plant was retained after two weeks. To meet the nutritional requirements, recommended dose of NPK (180 kg ha^−1^ of nitrogen, 140 kg ha^−1^ of phosphorus, and 90 kg ha^−1^ of potassium) was applied to each pot using urea, diammonium phosphate, and muriate of potash fertilizers. Phosphorus and potassium fertilizers were added as a base dose, while nitrogen was added in three splits: before sowing, after first watering, and when plants reached knee-high stage. Good-quality tap water was used for irrigation throughout the experiment. After 60 days of sowing, leaves were sampled to measure the RWC, chlorophyll *a*, *b*, carotenoids, and antioxidants (superoxide dismutase, catalase, ascorbate peroxidase, and malondialdehyde). At harvest (110 days after sowing), parameters related to growth and yield were recorded.

### 4.11. Determination of Relative Water Content

The RWC of maize leaves was assessed using Weatherley method [83]. Fresh, dry and completely turgid weights were considered. Following formula was used to calculate RWC.

RWC = FW − DW/FTW − DW

### 4.12. Estimation of Photosynthetic Pigments

Chlorophyll and carotenoid contents of freshly homogenized leaves were determined using acetone extract (80% *v*/*v*) by following the procedure described by Arnon [84]. For the measurement of chlorophyll *a*, *b*, and carotenoids, spectrophotometer (Thermo Electron Corporation, USA) was set at wavelengths 663, 645, and 480 nm, respectively. 

### 4.13. Extraction of Antioxidant and Oxidant Activities

0.5 g of fresh leaf tissue was used to analyze antioxidant enzymes. Fresh leaf tissue was pulverized in a cold bath, and mixed with a phosphate buffer (50 mM). Subsequently, mixture was centrifuged at 13,000 rpm for 24 min. Resulting supernatant was collected for further analysis of antioxidant levels. To determine the activity of ascorbate peroxidase (APX) activity, method outlined by Asada and Takahashi [85] was followed. Reaction mixture was prepared by combining 100 mL of enzyme extract, 0.5 mM ascorbic acid, 50 mM KH_2_PO_4_ buffer, and 0.1 mM H_2_O_2_. Similarly, blank solution was prepared without the enzyme extract. Absorbance of reaction mixture and blank was measured at 290 nm.

Catalase (CAT) activity was determined using the method described by Chandlee and Scandalios [86]. Assay mixture was prepared by combining 2.6 mL of KH_2_PO_4_ buffer (1 mM), 400 mL of H_2_O_2_ and 40 mL of enzyme extract. Breakdown of H_2_O_2_ in leaves was assessed by measuring light absorption at 240 nm. CAT activity was expressed as reducing 1 mM of H_2_O_2_ per minute per mg protein.

Activity of SOD was assessed by following the protocol outlined by Giannopolitis and Ries [87]. To prepare the reaction mixture, 0.2 mL of crude enzyme extract was combined with 50 mM phosphate buffer (pH 7.6), 13 mM methionine, 750 mM NBT, 4 mM riboflavin, and 0.1 mM EDTA. Absorbance of the reaction solution was measured at 560 nm using a spectrophotometer (Thermo Electron Corporation, USA). To measure the concentration of malondialdehyde (MDA), sample was homogenized in 0.1% trichloroacetic acid. Reaction mixture for this assay consisted of 2.5 mL of sample extract (0.5 mL), thiobarbituric acid (0.5% *w*/*v*), and trichloroacetic acid (20% *w*/*v*), and heated in a fume hood at 95 °C for 30 min, and then cooled in an ice bath. Absorbance at 532 and 600 nm was measured, and difference in absorbances (A532-A600) was used to calculate MDA concentration [88].

### 4.14. Growth and Yield Parameters

Shoot, root, and cob lengths were measured using a meter rod for three randomly selected plants per treatment. Total yield, 100-grain weight, and fresh and dried weight of the shoot, root, and cob were determined using an electronic balance. Before weighing, the shoot, root, and cob were sun-dried and subsequently dried at 60 °C for 48 h.

### 4.15. Sodium and Potassium Determination

Leaf samples were collected from plants, and preserved in polypropylene centrifuge tubes containing liquid nitrogen to maintain freezing temperatures. After thawing, frozen samples were crushed, and leaf sap was collected using a Gilson pipette. Subsequently, sap was subjected to centrifugation at 9000× *g* for 10 min, and resulting supernatant sap was analyzed for sodium (Na^+^) and potassium (K^+^) concentrations using a Flame Photometer following the methodology described by US Salinity Laboratory Staff [78]. The K^+^/Na^+^ ratio was determined by dividing the K^+^ values by Na^+^ values.

### 4.16. Statistical Analysis

Data were subjected to statistical analysis using two-way ANOVA with the statistical software Statistix 8.1. Tukey’s HSD test was employed to determine the differences in treatment means at a 5% probability level. Graphs illustrating the parameters’ mean and standard error (±SE) were generated using MS Excel. Additionally, Pearson correlation was conducted between various plant parameters, and a correlogram was constructed using the built “cor” function in RStudio and the “corrplot” package, following the instructions provided in R (R Core Team, Vienna, Austria, 2016).

## 5. Conclusions

Salinity poses a significant threat to global agricultural productivity and food security. Findings of this study demonstrated that salt stress adversely affects growth, biomass, yield, and RWC of maize plants while simultaneously inducing the accumulation of MDA and activities of CAT, SOD, and APX. Applying exogenous protectants, such as PGPR and SA, mitigated the harmful effects of soil salinity, resulting in improved plant growth, biomass, and yield. Present study concluded that exogenous application of PGPR and SA enhances osmotic adjustment, boosts the antioxidant defense system, and protects the photosynthetic pigment under salt stress, promoting overall plant growth. However, influence of ionic homeostasis on the protectant-induced defense response is relatively minor. Regarding evaluating parameters such as biomass, yield, and antioxidative activity, PGPR demonstrated greater effectiveness as a salt-stress protectant. Nonetheless, in several salt-stress parameters, the response of plants to SA treatment closely resembles that of PGPR. Furthermore, combined application of PGPR and SA proves more effective in conferring salt tolerance in maize plants than in their applications. Further research and field trials are needed to validate the practical applicability of these findings and provide reliable recommendations for farmers.

## Figures and Tables

**Table 1:** Physiochemical parameters of soil.

Parameters	Unit	Value
Textural class	---------	Sandy clay loam
pH	---------	7.71
Gravimetric moisture contents	%	34
ECe	dS m^−1^	1.46
Organic matter	%	0.64
Total Nitrogen	%	0.060
Available phosphorus	mg kg^−1^	6.91
Extractable potassium	mg kg^−1^	134.5

## Data Availability

Not applicable.
